# Predictors of the prolonged recovery period in COVID-19 patients: a cross-sectional study

**DOI:** 10.1186/s40001-021-00513-x

**Published:** 2021-05-06

**Authors:** SeyedAhmad SeyedAlinaghi, Ladan Abbasian, Mohammad Solduzian, Niloofar Ayoobi Yazdi, Fatemeh Jafari, Alireza Adibimehr, Aazam Farahani, Arezoo Salami Khaneshan, Parvaneh Ebrahimi Alavijeh, Zahra Jahani, Elnaz Karimian, Zahra Ahmadinejad, Hossein Khalili, Arash Seifi, Fereshteh Ghiasvand, Sara Ghaderkhani, Mehrnaz Rasoolinejad

**Affiliations:** 1 Iranian Research Center for HIV/AIDS, Iranian Institute for Reduction of High-Risk Behaviors, Tehran University of Medical Sciences, Tehran, Iran; 2 Department of Infectious Disease, Imam Khomeini Hospital, Tehran University of Medical Sciences, Blv. Keshavarz, Tehran, 1419733141 Iran; 3 Department of Clinical Pharmacy, Faculty of Pharmacy, Tabriz University of Medical Science, Golgasht St, Tabriz, 5166414766 Iran; 4 Department of Radiology, Imam Khomeini Hospital, Tehran University of Medical Sciences, Tehran, Iran; 5 Department of Clinical Pharmacy, Imam Khomeini Hospital Complex, Tehran University of Medical Sciences, Tehran, Iran

**Keywords:** COVID-19, Recovery, Time, Predictors, Dyspnea, Comorbidities

## Abstract

**Background:**

The clinical course of COVID-19 may vary significantly. The presence of comorbidities prolongs the recovery time. The recovery in patients with mild-to-moderate symptoms might take 10 days, while in those with a critical illness or immunocompromised status could take 15 days. Considering the lack of data about predictors that could affect the recovery time, we conducted this study to identify them.

**Methods:**

This cross-sectional study was implemented in the COVID-19 clinic of a teaching and referral university hospital in Tehran. Patients with the highly suggestive symptoms who had computed tomography (CT) imaging results with typical findings of COVID-19 or positive results of reverse transcriptase-polymerase chain reaction (RT-PCR) were enrolled in the study. Inpatient and outpatient COVID-19 participants were followed up by regular visits or phone calls, and the recovery period was recorded.

**Results:**

A total of 478 patients were enrolled. The mean age of patients was 54.11 ± 5.65 years, and 44.2% were female. The median time to recovery was 13.5 days (IQR: 9). Although in the bivariate analysis, multiple factors, including hypertension, fever, diabetes mellitus, gender, and admission location, significantly contributed to prolonging the recovery period, in multivariate analysis, only dyspnea had a significant association with this variable (*p* = 0.02, the adjusted OR of 2.05; 95% CI 1.12–3.75).

**Conclusion:**

This study supports that dyspnea is a predictor of recovery time. It seems like optimal management of the comorbidities plays the most crucial role in recovery from COVID-19.

## Background

Severe Acute Respiratory Syndrome Coronavirus 2 (SARS-CoV2) was first identified in China as the cause of a pandemic of pulmonary involvements [1]. The main route of viral transmission has been postulated to be through respiratory droplets and is thought to occur in close contacts [2]. Because of the possibility of underestimation of SARS-CoV2 infection, the seroprevalence evaluation has been conducted in the United States and has shown that infection rates may be up to 24 times higher than the reported [3]. The presence of risk factors including older age, hypertension, diabetes mellitus (DM), high d-dimer levels, male gender, severe hypoxia, and organ dysfunction has resulted in a worse prognosis in hospitalized patients [4].

A long duration of viral activity and shedding has been reported in studies, contributing to the virus’s high infectivity [5]. The most prevalent symptoms of COVID-19 have been described as fever and cough, which affect about 80% and 60% of the patients, respectively, and another commonly reported symptom was fatigue [6].

Studies in Chinese patients, early during the pandemic, reported that more than 50% of the patients had baseline comorbidities, including cardiac and metabolic diseases such as diabetes. Although dyspnea affected more than half of the patients, less frequently headache, productive cough, hemoptysis and diarrhea were also reported. Leukopenia, lymphopenia, increased prothrombin levels, d-dimer, lactate dehydrogenase and hepatic transaminases were significantly associated with severe disease in this study [7]. Besides, increased ratios of AST/ALT on hospital admission have been significantly associated with higher rates of mortality in COVID-19 patients [8]. Fever, cough and dyspnea are the most prevalent symptoms in some reports, and the presence of lymphopenia (defined as lymphocyte count ≤ 900/mm^3^), moderate ARDS or lack of treatment compliance could significantly predict the risk of mortality [9].

Less severe symptoms, including anosmia and ageusia, were reported in about 46% and 83% of the patients. These symptoms required a prolonged recovery period, and one-third of the patients did not recover during the study [10]. Further studies also reported that olfactory and gustatory symptoms are prevalent in COVID-19 patients, and they could remain persistent in more than 25% of the patients. They also reported a correlation between the duration of these symptoms and COVID-19 severity [11]. The prevalence of skin involvement in COVID-19 patients has been about 11%, with a different pattern [12]. Skin involvement due to COVID-19 might precede other manifestations in pediatrics and might occur due to hyperinflammation and coagulation disorders in the context of the disease [13]. Although vasculitis has been reported in COVID-19 patients, reliable evidence in this area is limited [14]. Besides features such as male gender and older age, it has been suggested that long term treatment with angiotensin receptor blockers (ARBs) increases the risk of SARS-CoV2 infection; however, the investigators recommended risk/benefit evaluation before changing practice [15].

In silico studies suggested that protease inhibitors such as lopinavir and darunavir and analogue nucleosides combination such as tenofovir and emtricitabine could be effective COVID-19 treatment regimen [16]. Nevertheless, no treatment changes the course of the COVID-19 completely. The World Health Organization (WHO) solidarity trial’s interim results did not report any significant benefit using lopinavir/ritonavir, Hydroxychloroquine, and interferon-β, although remdesivir improved outcomes in patients receiving low-flow oxygen [17]. In hospitalized COVID-19 patients receiving oxygen via mechanical ventilation or non-mechanical route, dexamethasone at a dose of 6 mg daily has been proven beneficial and diminish mortality significantly [18]. Recently, an open-label trial showed that the addition of tocilizumab to the treatment regimen of patients receiving high-flow oxygen or mechanical ventilation in the first 24 h of intensive care unit admission could lower the 90-day mortality rate [19].

The clinical course of COVID-19 may differ significantly in patients with distinct baseline characteristics. The disease course tends to last longer in older patients [20]. Older age, chronic cardiopulmonary disease, higher mean of interleukin-6 and d-dimer levels are proven related factors that increase hospital mortality rates of COVID-19 patients; median time to death, clinical deterioration, and the total duration of hospitalization has been reported 9, 3, and 33 days, respectively [21]. Based on one report, the mean duration of recovery time in COVID-19 patients was 25 days, and no difference in the recovery time was shown between males and females and patients older than 60 years and younger ones [22]. A similar clinical course of COVID-19 in patients older than 75 years compared to those younger has also been reported [23].

About two-thirds of the COVID-19 patients in outpatient settings return to their baseline health level after 2 to 3 weeks [24]. Although prolonged persistence of COVID-19 symptoms has been stated and fatigue, dyspnea, and cough were symptoms that lasted beyond 2 months, decreased quality of life has also been introduced to affect about half of the patients [25]. Other surveys also represented similar results in the outpatient setting; however, approximately 65% of these patients return to their usual health status after 7 days. The presence of hypertension, immunosuppression, and obesity delayed returning to a usual health condition in adults, with a direct relationship between the number of these comorbidities and the time needed to recover. Psychiatric disorders were also displayed to delay the time required to return to usual health status. The median time to symptom resolution reported being 4 to 8 days. The patients presented with fatigue, cough, and shortness of breath 2 to 3 weeks after the diagnosis [26].

The recovery period usually occurs around 10 days in patients with mild-to-moderate symptoms and about 15 days in critically ill or immunocompromised patients [27]. The findings in COVID-19 patients show that radiological improvement might begin 2 weeks after disease onset in patients who recover from the disease [28].

The data about the recovery time are limited and different factors might hasten recovery or delay it. Considering that in the current practice guidelines, symptoms are primarily the focus of isolation termination, determining a specific recovery period and related factors associated with early or delayed recovery could help plan the treatment course and predict the outcome in this group of patients. Therefore, this study was carried out to determine recovery time predictors in patients with COVID-19 among the Iranian population.

## Materials and methods

### Study design and subjects

This cross-sectional study was conducted between April and August 2020. All patients referred to the COVID-19 clinic of Imam Khomeini Hospital, a teaching and referral university hospital in Tehran, were evaluated for the study’s eligibility.

### Eligibility criteria

Patients with the highly suggestive symptoms of COVID-19 who had computed tomography (CT) imaging results with typical findings of COVID-19 according to radiologist report or positive results of nasopharyngeal COVID-19 RT-PCR were enrolled in the survey after signing a written informed consent form. The consent note was approved by the ethical committee of the Tehran University of Medical Sciences (TUMS). The patient examination and data registration were done by four physicians, and all CT imaging findings were verified by a radiologist. Patients evaluated in the clinic were admitted to the hospital if they had moderate to severe hypoxemia or unable to tolerate oral medication intake. All patients who refused to participate in the study or were unable to report the symptoms properly or could not be followed were excluded.

### Follow-up

Patients admitted to the hospital were regularly evaluated after discharge by five medical residents and followed up through telephone calls until the symptoms’ amelioration. The patients admitted to the ward were transferred to the intensive care unit (ICU) if they showed signs of worsening hypoxemia or developed any complications. In the outpatient setting, patients were followed by regular telephone calls and were admitted to the hospital in case of worsening symptoms or developing complications. All calls were made through an official hospital phone line. The patient’s phone numbers and identities were recorded and kept confidential per the hospital’s protocol and were not used without their permission. The recovery time was defined regarding the patient’s subjective statement indicating no symptoms than his/her baseline.

### Clinical data collection

Data regarding demographic variables, medical and medication history, laboratory data, place of admission, length of hospital stay, and recovery time were collected in specific forms for each patient. The main therapeutic option used in the outpatient setting was hydroxychloroquine, and medications prescribed for hospitalized patients were hydroxychloroquine, lopinavir/ritonavir (Kaletra), atazanavir (nonboosted with ritonavir or cobicistat), and in case of severe hypoxemia (defined as not attaining oxygen saturation of more than 88% with reservoir mask), corticosteroid was given.

### Statistical analysis

Data distribution was evaluated with Shapiro–Wilk test before the analysis. Data were elaborated as numbers on total (percentages), means ± standard deviations and median (IQR), based on their parametric and non-parametric distribution. Bivariate and multivariate logistic regression models were carried out to evaluate the significance of variables in determining the recovery time. Statistical analysis was conducted using IBM SPSS software, version 22.

The patient’s recovery period cut-off was based on the previous studies’ results [27]. The results of cohort studies were also used to determine the laboratory variables cut-offs [7].

Odds ratio (OR) and *p*-value for each factor were noted. The characteristics associated with the recovery time in the bivariate analysis at the *p*-value < 0.10 level were included as potential predictors. The final predictors retained those characteristics associated with the recovery time at the *p*-value < 0.05 level.

## Results

A total of 478 patients were enrolled by convenience sampling. One hundred twenty-six (26.3%) patients were managed in the outpatient setting, and ten patients were lost to follow-up because they were not willing to continue their cooperation or did not answer phone call after multiple attempts following the enrollment. Among the patients admitted, 19 (4%) of them were transferred to ICU. The patients’ mean age was 54.11 ± 5.65 years, and 44.15% of them were female. The median time to recovery was 13.5 days (IQR: 9, third quartile 20). Baseline characteristics of the enrolled patients are shown in Table 1.

**Table 1:** Baseline characteristics of the enrolled patients, Tehran, Iran, 2020

Variable	Report
Temperature °C (mean ± SD)	37.493 ± 0.77
Respiratory rate (breath/min) (mean ± SD)	20.09 ± 8.9
Pulse rate (beats/min) (mean ± SD)	88.02 ± 14.65
SpO2% (mean ± SD)	91.03 ± 5.67
White blood cell count (10^3^/mm^3^) (median, IQR) (*Q*_1_–*Q*_3_)	6.4, 4.2 (4.8–9)
Lymphocyte count (10^3^/mm^3^) (median, IQR) (*Q*_1_–*Q*_3_)	1100,743 (786–1529)
Hemoglobin (g/dL) (mean ± SD)	12.75 ± 2.215
Platelet count (10^3^/mm^3^) (median, IQR) (*Q*_1_–*Q*_3_)	218.02, 97.84 (178.16–276)
CPK (units/L) (median, IQR) (*Q*_1_–*Q*_3_)	119,125 (65–190)
ESR (mm/h) (median, IQR) (*Q*_1_–*Q*_3_)	72.7, 31.79 (65.21–97)
CRP(mg/L) (median, IQR) (*Q*_1_–*Q*_3_)	73.5,117.25 (30.75–148)
AST (units/L) (median, IQR) (*Q*_1_–*Q*_3_)	34, 21.75 (25.25–47)
ALT (units/L) (median, IQR) (*Q*_1_–*Q*_3_)	30,30 (10.75–49.75)
LDH (units/L) (mean ± SD)	628.58 ± 291.74
Creatinine (mg/dL) (median, IQR) (*Q*_1_–*Q*_3_)	1, 0.4 (0.8–1.2)
Medications	
Hydroxychloroquine	458 (95.6%)
Lopinavir/ritonavir	74 (15.4%)
Atazanavir	264 (55.1%)
Corticosteroids	54 (11.3%)
Duration of symptoms before admission (days) (median, IQR) (*Q*_1_–*Q*_3_)	7, 6 (4–10)
Length of hospital stay (median, IQR) (*Q*_1_–*Q*_3_)	
Ward (days)	4, 4 (4–8)
ICU (days)	10,7 (5.5–12.5)
Death	21 (4.4%)

ALT, alanine amino transferase; AST, aspartate amino transferase; CPK, creatinine phosphokinase; ESR, erythrocyte sedimentation rate; CRP, C-reactive protein; LDH, lactate dehydrogenase; SpO_2_, arterial oxygen saturation; *Q*_1_, first quartile; *Q*_3_, the third quartile

The bivariate analysis of the association of different clinical findings to prolonged recovery time is demonstrated in Table 2. The association between different laboratory and imaging studies and the prolonged recovery period is shown in Table 3. In bivariate analysis male gender (*p*-value 0.02), age older than 50 years (*p*-value 0.02), hypertension (*p*-value 0.005), DM (*p*-value 0.008), presence of fever (*p*-value 0.006), arterial O_2_ saturation < 93% (*p*-value 0.001), respiratory rate of ≥ 25 breaths/min (*p*-value 0.08), erythrocyte sedimentation rate (ESR) > 60 mm/h (*p*-value 0.05), and C-reactive protein (CRP) ≥ 91 mg/L (*p*-value 0.09) were significantly associated with prolonged recovery time. In multivariate analysis, only dyspnea had a significant association with the recovery time (*p*-value 0.02, the adjusted OR of 2.05; 95% CI 1.12–3.75). The results of the multivariate analysis are shown in Table 4.

**Table 2:** The relation between demographic and clinical findings and prolonged recovery period in bivariate analysis, in the enrolled patients, Tehran, Iran, 2020

Variables	Groups		OR (95% CI) Referent	*p*-value Referent
	Recovery time ≤ 14 days *N* (%)^*^	Recovery time ≥ 15 *N* (%)^*^		
Gender				
Male	161 (60.1%)	99 (49.5%)	1.54 (1.06–2.22)	0.02**
Female	107 (39.9%)	101 (50.5%)		
Admission location				
Outpatient	94 (35.1%)	31 (15.5%)	N/A	N/A
ICU	8 (3.0%)	11 (5.5%)	4.17 (1.54–11.30)	0.005**
Ward	166 (61.9%)	158 (79.0%)	2.89 (1.82–4.58)	< 0.001**
Age group (years)				
≤ 50	117 (49.0%)	63 (37.1%)	1.63 (1.09–2.43)	0.02**
> 50	122 (51.0%)	107 (62.9%)		
O_2_ saturation %				
≥ 93	138 (53.1%)	64 (32.5%)	2.35 (1.60–3.46)	< 0.001**
< 93	122 (46.9%)	133 (67.5%)		
Pulse rate (beats/min)				
60–99	151 (61.4%)	108 (55.7%)	1.27 (0.86–1.86)	0.23
≥ 100	95 (38.6%)	86 (44.3%)		
Respiratory rate (breaths/min)				
< 25	220 (84.0%)	152 (77.6%)	1.52 (0.95–2.43)	0.08**
≥ 25	42 (16.0%)	44 (22.4%)		
Temperature (°C)				
≤ 37.2	116 (43.6%)	86 (43.7%)	0.10 (0.69–1.45)	0.99
≥ 37.3	150 (56.4%)	111 (56.3%)		
Coronary artery disease (CAD)	36 (13.4%)	36 (18.0%)	1.42 (0.86–2.34)	0.18
Hypertension	61 (23.0%)	70 (35.0%)	1.80 (1.20–2.71)	0.005**
Diabetes mellitus	61 (22.8%)	68 (34.0%)	1.74 (1.16–2.62)	0.008^**^
Chronic obstructive pulmonary disease	17 (6.3%)	13 (6.5%)	1.03 (0.49–2.17)	0.95
Immune compromised	34 (12.7%)	25 (12.5%)	0.98 (0.57–1.71)	0.95
Chronic liver disease	5 (1.9%)	2 (1.0%)	0.53 (0.10–2.77)	0.45
Chronic renal failure	9 (3.4%)	12 (6.0%)	1.84 (0.76–4.45)	0.18
Cancer	22 (8.2%)	20 (10.1%)	1.25 (0.66–2.36)	0.49
Pregnancy	1 (0.4%)	2 (1.0%)	2.69 (0.24–29.84)	0.42
Smoking history	15 (5.6%)	11 (5.5%)	0.98 (0.44–2.19)	0.96
Opium history	3 (1.1%)	2 (1.0%)	0.89 (0.15–5.39)	0.90
Dyspnea	135 (50.4%)	135 (67.5%)	2.05 (1.40–2.99)	< 0.001**
Fever	143 (53.4%)	132 (66.0%)	1.70 (1.16–2.48)	0.006**
Headache	78 (29.1%)	52 (26.0%)	0.86 (0.57–1.29)	0.46
Sore throat	47 (17.5%)	32 (16.0%)	0.90 (0.55–1.47)	0.66
Chills	128 (47.8%)	104 (52.0%)	1.19 (0.82–1.71)	0.36
Sputum	5 (1.9%)	10 (5.0)	2.77 (0.93–8.23)	0.07**
Sweating	46 (17.2%)	43 (21.5%)	1.32 (0.83–2.10)	0.24
Myalgia	151 (56.3%)	132 (66.0%)	1.50 (1.03–2.20)	0.04**
Runny nose	18 (6.7%)	9 (4.5%)	0.65 (0.29–1.49)	0.31
Cough	176 (65.7%)	148 (74.0%)	1.49 (0.99–2.23)	0.05**
Sneezing	10 (3.7%)	10 (5.0%)	1.36 (0.55–3.33)	0.50
Nausea and vomiting	53 (19.8%)	62 (31.0%)	1.82 (1.19–2.79)	0.006**
Diarrhea	30 (11.2%)	20 (10.0%)	0.88 (0.49–1.60)	0.68
Stomach ache	26 (9.7%)	18 (9.0%)	0.92 (0.49–1.73)	0.80
Hemoptysis	4 (1.5%)	13 (6.5%)	4.59 (1.47–14.29)	0.009**
Chest pain	65 (24.3%)	57 (28.5%)	1.25 (0.82–1.89)	0.30
Vertigo	35 (13.1%)	16 (8.0%)	0.58 (0.31–1.08)	0.09**
Dizziness	29 (10.8%)	19 (9.5%)	0.87 (0.47–1.59)	0.64
Joint pain	33 (12.3%)	42 (21.0%)	1.89 (1.15–3.12)	0.01**
Generalized weakness	109 (40.7%)	100 (50.0%)	1.46 (1.01–2.11)	0.05**
Sleep disorder	18 (6.7%)	11 (5.5%)	0.81 (0.37–1.75)	0.59
Anosmia	33 (12.3%)	27 (13.5%)	1.11 (0.64–1.92)	0.70
Ageusia and dysgeusia	24 (9.0)	24 (12.0)	1.39 (0.76–2.52)	0.28
Intubated	9 (3.4%)	47 (23.5%)	8.84 (4.22–18.54)	< 0.001**
Corticosteroids	17 (6.3%)	36 (18.0%)	3.24 (1.76–5.96)	< 0.001**
Hydroxychloroquine	167 (95.9%)	168 (99.4%)	7.042 (0.85–57.86)	0.069
Lopinavir/ritonavir (kaletra)	34 (19.5%)	36 (21.3%)	1.115 (0.65–1.88)	0.686
Atazanavir	119 (68.4%)	128 (75.7%)	1.443 (0.89–2.32)	0.130

*ALT* alanine amino transferase, *AST* aspartate amino transferase

* Subgroups do not always add up to total due to missing data

** Statistically significant

**Table 3:** The relation between laboratory and imaging findings and prolonged recovery period in bivariate analysis, in the enrolled patients, Tehran, Iran, 2020

Variables	Groups		OR (95% CI) Referent	*p*-value Referent
	Recovery time ≤ 14 days *N* (%)^*^	Recovery time ≥ 15 *N* (%)^*^		
Normal CT scan				
Yes	17 (8.8%)	4 (2.5%)	3.75 (1.23–11.37)	0.02**
No	177 (91.2%)	156 (97.5%)		
Lactate dehydrogenase (units/L)				
< 480	29 (37.2%)	35 (30.4%)	1.35 (0.74–2.48)	0.33
≥ 480	49 (62.8%)	80 (69.6%)		
Creatine phosphokinase (units/L)				
< 195	44 (75.9%)	75 (78.9%)	0.84 (0.39–1.82)	Referent
≥ 195	14 (24.1%)	20 (21.1%)		0.66
AST (units/L)				
< 41	63 (63.6%)	84 (67.2%)	0.85 (0.49–1.49)	0.58
≥ 41	36 (36.4%)	41 (32.8%)		
ALT (units/L)				
< 41	63 (64.9%)	86 (68.8%)	0.84 (0.48–1.48)	0.55
≥ 41	34 (35.1%)	39 (31.2%)		
Erythrocyte sedimentation rate (ESR) (mm/h)				
≤ 60	57 (41.6%)	44 (30.1%)	1.65 (1.01–2.70)	0.05**
≥ 61	80 (58.4%)	102 (69.9%)		
C-reactive protein (CRP) (mg/L)				
≤ 90	96 (62.3%)	83 (52.9%)	1.48 (0.94–2.32)	0.09**
≥ 91	58 (37.7%)	74 (47.1%)		
Platelet (cell/mm^3^)				
< 150,000	41 (24.7%)	39 (23.6%)	0.94 (0.57–1.56)	0.82
≥ 150,000	125 (75.3%)	126 (76.4%)		
White blood cell (WBC) (cell/mm^3^)				
< 4000	26 (15.8%)	23 (14.1%)	0.10 (0.54–1.85)	0.99
4000–9999	117 (70.9%)	103 (63.2%)	1.90 (0.88–4.11)	0.10
≥ 10,000	22 (13.3%)	37 (22.7%)		
Lymphocyte count (cell/ mm^3^)				
≤ 1000	56 (44.4%)	64 (46.4%)	1.08 (0.67–1.76)	0.75
> 1000	70 (55.6%)	74 (53.6%)		

*ALT* alanine amino transferase, *AST* aspartate amino transferase

*Subgroups do not always add up to total due to missing data

**Statistically significant

**Table 4:** Independent association with recovery time among patients with COVID-19 in multiple conditional logistic regression; Tehran, Iran, 2020

Variable	AOR (95% CI)	*p*-value
Gender		
Male	Referent	Referent
Female	1.48 (0.84–2.62)	0.18
Age (years)		
< 39	Referent	Referent
39–49	0.98 (0.39–2.46)	0.97
50–59	0.81 (0.31–2.08)	0.66
60–69	0.70 (0.27–1.81)	0.46
≥ 70	0.82 (0.29–2.36)	0.71
Hypertension	1.20 (0.64–2.26)	0.56
Diabetes	1.40 (0.73–2.66)	0.31
Dyspnea	2.05 (1.12–3.75)	0.02
Fever	1.00 (0.57–1.76)	0.99
SpO_2_	1.11 (0.60–2.05)	0.74
Respiratory rate	0.97 (0.46–2.05)	0.94
ESR	1.45 (0.78–2.71)	0.24
CRP	1.34 (0.75–2.41)	0.33

## Discussion

In our study, multiple related factors could prolong the recovery period in patients with COVID-19 in the bivariate analysis, but dyspnea might be the only predictor that significantly affects the recovery duration. It is among the symptoms that long persist following initial recovery from COVID-19 [25]. Dyspnea has been reported more frequently in patients not surviving from COVID-19 compared to those who survive (*p* < 0.001) [29]. In addition to worsening the prognosis of the disease and increasing the possibility of death, dyspnea, especially with minimal exertion, necessitate hospital admission and specialized care and is therefore expected to prolong the disease course [30, 31]. A higher prevalence of dyspnea in patients with severe or critical COVID-19 has been represented in other studies as well [32]. The final model in our study showed that the presence of dyspnea at baseline could significantly increase the time needed for recovery from COVID-19, and this increase the prognostic value of this symptom and shows that besides the increased possibility of death and severe disease, patients with dyspnea are expected to recover later than those without this symptom. None of the other symptoms evaluated in the current study demonstrated a prognostic value, although they might help predict other disease outcomes or complications.

In one meta-analysis, it has been proved that the male gender could be related to the significant increase in intensive care admission or death due to COVID-19 (ORs of 1.39 and 2.84, respectively) [33]. The lower rate of COVID-19 symptoms and higher survival rate observed in females has been attributed to higher estrogen levels in women of reproductive age, although no significant difference was reported between males and females regarding the recovery time [34].

No difference has been reported in recovery time between males and females of any age group in some studies, although the incidence of disease was lower in females of 20 to 70 years [22]. In contrast, some studies postulated that female patients might recover more quickly compared to males [35]. The results of this study depicted that although the male gender had a significant role in prolonging the recovery period in bivariate analysis, there was no significant difference between males and females of any age in the recovery period in multivariate analysis, and these findings agree with the previous studies [35]. These data suggest the need for further studies to evaluate the real effects of gender on the recovery from COVID-19.

The hospital admission site is a known factor that could affect the length of hospital stay and prolong the recovery period. Previous studies reported a higher length of hospital stay and mortality in the patients admitted to the ICU than those admitted to the ward [36]. The hospital admission, ward or ICU, or outpatient site did not significantly contribute to the recovery time prolongation in the current study, as is demonstrated in the final analysis. It could theoretically magnify the importance of optimal care of the patients that the primary need for intensive care might not significantly contribute to longer the recovery period.

Older age is considered a risk factor for mortality [37]. The younger patient’s recovery rate is significantly higher than those older than 70 years [38]. Patients between 1 and 24 years recover more rapidly than those older than 24 years [39]. Our study showed no difference in the recovery time of patients younger or older than 50 years in the final model; however, a significant relationship was noticed in the primary analysis. While younger age might be a predictor of a higher recovery rate, it might not significantly contribute to faster recovery. Previous data that illustrated faster recovery in younger patients mainly reported such findings in patients younger than those evaluated in our study as the mean age of the patients in our study was 54.1 years. Previous studies have reported that the highest rates of disease incidence were seen in patients older than 50 years; therefore, no faster recovery in younger patients might be generalizable to all COVID-19 patients, and our data might be of more value because it evaluated the relationship in a meaningful age cut-off [40].

Coronary artery disease could result from COVID-19 infection, and its presence has been reported to increase the mortality rate in these patients [41, 42]. Although CAD could increase the COVID-19 complications, it is not supposed to prolong the recovery time as it is presented in the results of this study that CAD did not significantly contribute to increasing the recovery period longer than 15 days.

Hypertension could significantly prolong the duration of disease recovery. Hypertension has been reported to worsen COVID-19 outcomes and increase the ICU admission rate [43]. Optimal management of hypertension seems to be the main factor affecting the patients’ outcomes with COVID-19, as shown in our study.

Early studies in patients with DM and COVID-19 illustrated worse outcomes and more severe disease [44]. In COVID-19 patients with DM, besides the female gender and high platelet mean volume, the use of insulin could significantly be a predictor of recovery [45]. Although DM could be a predictor of disease severity or worse outcome, our results showed that in patients recovering from COVID-19, the presence of DM is not considered a significant determinant of the recovery period.

Fatigue, expectoration, and stuffed nose could significantly predict the disease’s severity in COVID-19 patients [46]. The longer duration of fever, a common symptom in patients with COVID-19, was related to a higher possibility of ICU admission, and the presence of high fever has been associated with a high rate of acute respiratory distress syndrome (ARDS) and a lower rate of death [47, 48].

The association between high serum levels of CRP and ESR and COVID-19 progression and severity of the disease is known [49, 50]. Our results did not present a significant relationship between these biomarkers and the time needed for recovery; these biomarkers could help classify patients based on their disease severity and prognosis.

This study failed to prove an association between corticosteroid receipt and time to recovery. In the previous reports receiving systemic corticosteroids have been suggested as a risk factor for extended viral shedding [31]. On the other hand, the use of dexamethasone has been shown to lower the mortality rate in hospitalized COVID-19 patients receiving supplemental oxygen [51]. Although corticosteroids’ effects on mortality are well known based on the current data, their effects on time to recovery need further studies and the current results show no significant association [52].

Intubation and mechanical ventilation are required to manage COVID-19 patients with severe hypoxemia or acute respiratory syndrome. Patients who required invasive mechanical ventilation due to COVID-19 had a prolonged hospitalization duration and poor outcomes [53]. Although the need for mechanical ventilation could predict the risk of mortality and prolonged hospitalization, our survey results showed that it could not predict the recovery time. The overall number of patients who received invasive mechanical ventilation in our study was limited, lowering the results’ reliability.

## Limitations

Our findings are subject to several limitations. The criteria for recovery were based on the subjective statement, and this could have resulted in conclusion errors; inviting patients to the clinic for evaluation could have been a more valid option to confirm recovery. Not all the enrolled patients had positive results of the RT-PCR test, but a multidisciplinary team reviewed all the data regarding COVID-19 diagnosis, and only patients with the nearly proved disease were enrolled in the investigation. Overall, 35 patients received antibiotics; because of practical limitations, the exact data about bacterial co-infection could not be provided, and this could have influenced recovery time; however, the total number of patients who received antibiotics was limited.

## Conclusion

The presence of dyspnea in COVID-19 patients could significantly increase the recovery time. The final model failed to show any significant relationship between other factors and the time to recovery. It seems like optimal management of the comorbidities plays the most crucial role in COVID-19 recovery.

## Data Availability

Data regarding the study process and other confirmatory information may be available upon request.
